# New York Odontological Society

**Published:** 1891-04

**Authors:** 

**Affiliations:** New York Odontological Society


### Reports of Society Meetings.

### NEW YORK ODONTOLOGICAL SOCIETY.

The New York Odontological Society held its regular meeting Tuesday evening, January 20, 1891, at the Academy of Medicine, No. 17 West Forty-third Street.

The President, Dr. William H. Dwinelle, in the chair.

### INCIDENTS OF OFFICE PRACTICE AND CASUAL COMMUNICATIONS.

*Dr. S. G. Perry.*—Mr. President, I do not want to take up the time of the essayist of the evening by a consideration of trifling things, but as the sum total of our knowledge is made up of trifling things, perhaps I may be allowed a minute or two to bring to your notice some instruments I have recently had made.

At a recent meeting of this Society I showed some root-canal reamers that I ventured to call "safety" reamers.

Their safety consisted in having their cutting-flanges very shallow, so that they would not bite nor wedge, and their handles very small, so that even in careless hands but little purchase could be had on them.

They were made of forty-two-gauge wire, in lengths of from half an inch to four inches and a half, and the short ones were to be used in the back-action hand-piece, or to be rotated between the thumb and finger.

The longer ones had handles only half an inch long, and were intended to be rotated between the thumb and finger, or in the engine hand-piece.

Constant use of these reamers has so impressed me with their value that I have gone a step farther and had some of them made with long handles, to be used in the hand in the ordinary way or to be used in the engine if desired.

These instruments are seven inches long and the handles four and a half inches. The shafts are very flexible, and they can be easily rotated while bent at right angles.

The ends of the handles are smooth, so as to rotate easily in the palm of the hand, and that part of the handle grasped by the thumb and finger is slightly milled, so as to give a little more control of the instrument.

The cutting-points are long and have a very gradual taper, which adds to their safety, as they cannot cut a great deal in one place. They do not cut rapidly, which is another element of safety.

Some of the points are extremely small, and for opening the end of a root, in order to tap an abscess, are superior to any instruments I have ever seen.

I have never yet known one of them to break.

In showing these instruments, I do not want to be understood as advocating the reaming of root-canals to any great extent. Many roots are flat, and all are not straight, and generally it is a dangerous practice, particularly when the engine is used; but most canals can be opened a little to advantage, and the gradual taper of these instruments makes them admirably suited for that purpose.

As before stated, the cutting-blades are shallow, and on each instrument there are a relatively large number of them, so that they do not bite too much, or bind so as to endanger the instrument. The blades are cut slightly spiral so that the edge of each presents to the greatest advantage.

They are not intended to be self-cleansing. To make them so would necessitate deeper channels and higher blades, and that would make an instrument that would bite so deeply that in the engine or in a careless hand it might break.

But the instrument can be often removed from its work, when it will free itself and be ready for a new start.

*Dr. S. E. Davenport.*—About a year ago a lady patient presented herself in great distress of mind, and in need, she claimed, of immediate attention. Upon examination I found that the entire enamel face of the right superior central had split off from the rest of the tooth, the break extending as far as the enamel did, but not quite to the gum,—all the gums having receded somewhat. The canal and pulp-chamber of the tooth had been filled with oxychloride of zinc for several years, and large gold fillings had formed both distal and mesial surfaces of the tooth until the break dislodged them.

The tooth seemed to invite the misfortune, for, besides the weak features already referred to, the cutting-edges of all the incisors were much worn, and the anterior and posterior plates of enamel were therefore separated at the very point where the greatest strength was needed, for the patient was obliged to use the front teeth more or less in masticating, several molars having been lost for probably ten or fifteen years. What was to be done?

The first thought, of course, was a porcelain crown, but, aside from the voiced objections of the patient, there were other reasons why it did not seem best at that time to resort to that method. The teeth being crowded, of a very peculiar color, and singularly constricted at their necks, I doubted my ability to find a suitable porcelain tooth to attach to the root, and it always seems a pity to cut off a natural crown, or the strong parts of one, unless it be really the last resort.

The enamel face had only slight portions of dentine attached to it, and certainly seemed too frail to do much with, but when placed in position, with the exception of the sides from which the gold fillings had fallen at the time of the accident, the tooth certainly looked well enough to encourage me in at least experimenting with the parts remaining.

A narrow band of twenty-two-carat gold was carefully fitted around the neck of the tooth, extending from slightly below the gum to a trifle beyond the point of fracture.

The measurements for this band were taken with the enamel face in position, and, when soldered, I was able, by slightly pinching the band together, to get it past the portion of the crown which was attached to the root, there being so much of this structure wanting where the proximal fillings had been. With sufficient force I adapted the band to its place, its front lower edge then being ready to receive the upper portion of the enamel face.

The dam was then applied to all the front teeth, the remaining portions of the broken crown were slightly grooved, and the enamel face was cemented to its proper place with Smith's adamantine cement, the cement also forming the fillings of the approximal surfaces and running through the groove at the cutting-edge. When this had hardened somewhat, a small hole was drilled through the centre of the enamel face and extended through the tooth to its palatine surface. A rivet of gold and platinum, fitting the drill-hole quite closely, was cemented into this shaft and polished off flush with the enamel on both anterior and posterior surfaces.

A few days since I saw the patient for other operations and could detect no weakness anywhere about this tooth, though the zinc phosphate has worn away slightly at the cutting-edge and may need occasional renewal.

The band which holds the upper edge of the fractured enamel seldom shows, but if the lip is drawn up the band looks, at a distance of three feet, like a marginal filling.

The rivet is seen only if looked for, and the patient is well pleased with the result of the experiment.

*Dr. N. W. Kingsley.*—I do not suppose, Mr. President, that Dr. Davenport would pretend that that was the first time an operation of that kind had been performed, but only presents it as a successful case of that sort. Not less than fifteen years ago a gentleman who was at that time Secretary of State of the United States came into my hands, having one of his central incisors split off in exactly the same way, and it was treated in the same way that Dr. Davenport describes, with the exception that I did not put a band around the neck of the tooth; but I riveted and cemented it in the same way. It lasted about a year and a half and then split in two; but during that year and a half it was the best thing that could have been done.

*Dr. Perry.*—I have a lady patient who wears five of those restorations. Three or four have been worn four or five years at least. The pieces were simply dovetailed and cemented on with zinc phosphate in the usual way. Four or five years ago a lady came down from White Plains with the whole outer plate of enamel of a central broken off. She brought the piece in her pocket-book. I dovetailed the piece and set it on with zinc phosphate, and it has not come off yet. I am glad to hear Dr. Davenport report this case, because I believe it is better to save a natural tooth in that way than to attempt too soon to crown it. Crowning can always be done as a last resort.

*The President.*—I saw in Dr. Perry's office, the other day, a patient for whom he had cemented a piece to a tooth, which, if I mistake not, he had implanted.

*Dr. Perry.*—Yes, that was an implanted tooth. It was an old tooth, and having been out of the mouth for some time it was dry and full of cracks, and one day the patient came to me in great trepidation with a V-shaped break out of the end of the tooth. It involved the inner and outer plate of enamel, and extended from the cutting-end of the tooth perhaps half-way up to the gum. It gave the mouth a singular appearance, the tooth seeming to be divided into two pyramidal spikes. I very quickly and easily cemented the piece in its place with the oxyphosphate, and it has been on now, I think, a little over a year and has not changed its position at all. Those who have had much experience in implanting teeth must have learned, to their discouragement, that teeth that have been out of the mouth for some time become thoroughly dry and liable to crack, particularly the older teeth. Young teeth do not seem to crack so readily. I have had such an unfortunate experience in implanting teeth in that way that if I cannot obtain a reasonably perfect tooth I implant a perfect natural root, and put on that root an artificial crown. Not long since I planted a root in a new socket, and while the root was in place filed the end of it and ground and fitted a crown. I then took the root out and attached the crown with a pivot in the usual way, and, setting it back, ligatured it with silk to the tooth adjoining. I told the patient to report in a day or two. She did not come for several days, and I finally sent my secretary to inquire the reason, and she said she had been so comfortable she had not thought it necessary to call.

*Dr. William Jarvie.*—I have repaired quite a number of broken bicuspids in a little different manner from that which has been reported; cases in which the outer, or buccal, cusp has been broken off, the remaining part of the tooth being strong. I have taken either a cuspid-plate tooth or a tooth for rubber, ground grooves in the porcelain, or, where practical, have utilized the pins, and attached this to the portion of the natural crown remaining, with zinc phosphate. I have quite a number of such cases that have been doing service for several years. Two I saw the other day that have been on ten or twelve years, and had not been disturbed at all.

*The President.*—Gentlemen, I have the pleasure of introducing Dr. George T. Stevens, of this city, who will read a paper entitled "Some Conditions of Mutual Interest to the Dentist and the Oculist."

(For Dr. Stevens's paper, see page 145.)

*The President.*—Gentlemen, we have listened to a remarkably interesting paper this evening, and one that has very intimate relations to our profession, on reflex action. Attention has been called to this question very frequently of late. We have certainly been highly entertained. The subject is open for discussion.

*Dr. Kingsley.*—Dr. Stevens queries if I may not have changed my mind during the time that has elapsed since the quotations made from my book were written. I have not changed my mind, and I am half inclined to think I wrote wiser than I knew. But I believed it then, and I believe it still. I have seen nothing in the fifteen years that have elapsed since that passage was written (which was not published in book form until some years after) to change my mind. I infer from the opinions given by the essayist this evening that he adopts and confirms the same doctrine. I

cannot quite see how any one who has made any extended observations of those matters and reflected upon the subject can come to any other conclusion.

There is a subject akin to this, germane to it, which you will permit me to speak upon a few minutes. It has been broadly hinted at by the essayist, and was brought particularly before us at our meeting last December, when we were discussing the subject of the influence of adenoid growths upon the form of the jaw or the palate. I have seen a great deal of malformations of the palate, using the term palate in the broader sense, malformations of the maxillæ on each side of the palate, the *tout-ensemble;* I have been puzzled a great deal to find a reason for it; and in the absence of finding a specific cause I have fallen back upon the general one embodied in the statement which I made so many years ago. But particularly since that paper was read, two months ago, has my attention been called to the subject in the cases of three patients who are in my hands now for treatment; these three cases are similar, and distinctive from any other I may happen to have. All three have protruding upper teeth. The teeth are standing forward, not all of them exactly alike, but in each the teeth protrude so much that it is difficult for the lips to be brought together, and when the mouth is open, as in speaking, the teeth are in full view. One of these patients is the daughter of a distinguished physician in this city. She is now thirteen years of age. She has been under the care of a physician, who removed from her upper pharynx an adenoid growth. It is stated that before that time she always breathed through the mouth, never through the nose. Now she breathes through the nose very well, but there is a deformity of the jaw. I took models of that case. Only the last teeth, the second molar on each side above and below, occluded. The jaws were open in front fully half an inch, and the upper teeth were standing forward. By a cursory examination I got the idea that the lower jaw was at fault, that it was bent down, but on taking models of the case and articulating them, there was as perfectly formed a lower jaw as I ever saw,—symmetrical in proportions and symmetrical in profile. The superior jaw, from the canine region back, dropped, and the dropping of the teeth manifested itself particularly at the locality of the twelve-year-old molar teeth, and that kept the teeth in front from coming together, leaving a space of a half to three-quarters of an inch between them. There is no question about the upper teeth protruding; there was not only a sinking of the upper jaw at the sides, but the front teeth protruded. The upper molar teeth stood directly over the lower ones and articulated properly with them, so far as the perpendicular line was concerned. The palatal arch was broad, not high, not narrow, but what is called a saddle-shaped arch. Notwithstanding the fact that there had been adenoid growths there, and the fact that the child had been a mouth-breather, it does not seem to me possible that it is other than a mere coincidence, or that this form of jaw is the certain result of those adenoid growths. That jaw was perfectly formed in infancy; the development of the teeth was perfect as to line; it was only when the permanent teeth were erupted that the deformity began to show itself. I cannot see how a growth in the upper pharynx is going to bulge down,—not the palatal bone, but the alveolar process at the side, right under the antrum.

The other two cases came into my hands about the same time. One of them was the daughter of a physician in Cincinnati, a man of education and standing. He brought his daughter to me; he heard through Dr. Lincoln, with whom he had a personal acquaintance, something about this question of adenoid growths, and he took a deep interest in the matter. Here was the projecting jaw of his daughter; the jaw projected a great deal more than the other cases that I have described, and more than any other case that I ever saw. That child is thirteen years of age; there was no adenoid growth, and she had never been a mouth-breather. Her lower jaw was so nearly a copy of the one I first described that I have had the models mixed two or three times, and it was difficult to distinguish them. There is a malformation of the upper jaw, but no adenoid growth.

In the third case there was a projection of the front teeth. There was some evidence to my mind, in my early practice, that this condition was due to thumb-sucking, or to sucking something, but I begin to doubt that somewhat. This third case presented all the evidences of thumb-sucking, but I found on inquiry both from the mother and the boy himself that there had never been any thumb-sucking. I also found there was no adenoid growth. Now, when we come to V-shaped or projecting jaws, I do not think, if I had not already put myself on record in regard to their origin, I would dare to do it now. Why? Because it seems to me that the more I see, the less I know, the more befogged I am, and the less I am able to give any scientific explanation of the real causes that produce these results.

*Dr. Perry.*—I was extremely interested in this excellent paper, in which the subject was so clearly and so plainly stated, so free from technical words and yet so full of good sense. There can be no consideration so important to a dentist as the proper care of his eyes. I am certain that the poorest professional work I have ever done was that which was done four or five years ago, before I suspected there was any need for the use of glasses. My attention was called to the matter by a remark made by Dr. Jack, who said he was certain that dentists often suffered from not early being made aware of the need of glasses. I am certain that in my case I do better work now than I did for some time before I put them on. The idea that I did not yet need glasses was confirmed by the oculist to whom I went to have my eyes examined, for he said, "Your eyes are perfect; what did you come to me for?" I repeated the remark that Dr. Jack had made, and the oculist said I might put them on without harm if I wanted to, but he hardly thought I needed them yet. I did put them on, and, although so early, I am satisfied that from that time I have done better work than I was doing, and with less danger of straining the eyes. This last consideration is one of the greatest reasons for using glasses early. Very severe headaches have been very common with me for a great many years. I cannot say whether they were more frequent or more severe before I wore glasses than they have been since, but I am inclined to think, after hearing Dr. Stevens's paper to-night, that perhaps the best solution of the cause of my headaches is very close application to work the day before and late reading at night. I have sometimes thought I could trace a connection between very close application to my work on a dark afternoon and a very severe headache the following morning. The life insurance people have told me that I am in perfect health, and yet for years I have had these dreadful headaches at quite frequent periods, and I have never before been able to assign a cause that now seems so reasonable as that of overstraining the eyes.

*Dr. G. W. Weld.*—This paper has been very interesting and instructive. In relation to the wearing of glasses, I want to say that two or three years ago a gentleman came to me to have his teeth attended to, and incidentally said he was suffering, or had suffered, with intense headaches; also that he had some trouble with his eyes. I asked why he did not go to some oculist and have his eyes examined. He said his eyes did not trouble him very much, and he had not thought very much about glasses, but he would go. I believe he went to Dr. Agnew, who prescribed certain glasses, and afterwards told me he had a pronounced case of astigmatism. I have seen the gentleman within a short time, and he informed me that very soon after he had his eyes examined and the glasses adjusted his headaches left him, and he now has no suffering whatever, so far as his head is concerned. It seems to me, as Dr. Perry has just said, that it is better to use glasses a little too early than a little too late.

Now, with reference to the paper and the relations between the eyes and the teeth, it seems to me that the mutual benefit, so to speak, is rather on the side of the dentist, for while the oculist can, by a process of elimination, determine the cause to be reflex disturbances, due to some affected tooth, yet I do not see how the dentist can tell just exactly when that reflex disturbance occurs. When I recommended the gentleman to Dr. Agnew I did it more out of sympathy than knowledge. I did it on general principles. I did not do it because I saw or understood there was a reflex disturbance.

*Dr. William F. Holcombe.*—I have known Dr. Stevens for many years, and he has a specialty within a specialty. Those anomalies of vision, as they are called,—squint-eye and insufficiencies of action of the eye-muscles,—are not always relieved by glasses. It has been recognized for a great many years that such cases could be benefited for a short time only with glasses; then patients have to change to other glasses, and finally no kind of glasses will suit. In this line of eye-diseases Dr. Stevens excels, and has been in advance of other oculists in relieving the irritation that comes from these defects of muscular action, or want of accommodation of the muscles of the eye. If any gentleman present will try to look like this,—squint,—he will see that it will cause pain in the brain. Suppose that goes on for twenty or thirty years! But there is Ben Butler, who has been doing it all his lifetime; and I have often wondered if he has constant headaches. There are thousands of people who have headaches on account of squint-eyes, but many people squint and have no headaches at all. I have sent many patients to Dr. Stevens that I could not benefit, and I have never known of one whom he has not helped. So I came here to learn; and I want some of these doctors to give some explanation how, by overuse of the eye, a man has the *toothache*, as Dr. Stevens has just stated he had. I want some of these doctors who must know of such *similar cases* to relate them. I have not been able to solve that from anything I have seen or heard, or any cases excepting the one that Dr. Stevens has given us. I understand how headaches will come from not being able to see correctly, or how they can come from overuse of the eyes; but I cannot quite see sufficient connection between the nervous structure of the eye and the teeth that would account for such pain in teeth. I feel very grateful to Dr. Stevens, and think the American oculists ought to recognize him as being far in advance in his specialty. He read a paper two or three years ago in Washington before the Ophthalmological Congress, and I then believed he was so far ahead of them that they did not appreciate his paper. I do not think they understand the skill and ability which he has succeeded in obtaining from his constant performance of operations upon these very peculiar cases that he has been treating for thirty years. That skill does not come in a moment. It is a very difficult thing to cut an eye just enough and no more. One can cut a squint-eye very easily, but often it is found that the eye turns out just as much as it turned in before the operation, because it has been cut too much. I would rather operate for cataract than for strabismus. I think it one of the most difficult operations to do correctly and exactly, because squint-eyes come very often from the eye not being able to see well. The eye turns because it is not used, and I know of a dozen cases of that kind within thirty-five years, where the squint has only existed because of permanently defective vision.

The first man to cut an eye for squint in this country is living to-day; I think it is Dr. Detmold. It is only a short time that this operation has been performed generally in the profession. It is only recently that we have become at all familiar with these delicate operations for changing and correcting the deficiencies of vision arising from irregular action of the eye. How can we come to a correct conclusion in this matter? One man who consulted me for great pain in his head was the eminent engraver, Edward C. Marshall. He overworked with one eye, as engravers and jewellers do, and the pain in his head was doubtless due to the use of one eye, and the result of the use of one eye upon the brain. Rest alone cured him.

Mr. President, I want to ask this Society about a small matter. During the war I had a pupil who went down to South Carolina, and among the things that he brought home was a set of dental instruments of very antique date; and I would like to know if there is any place where I can deposit them, where they would be of interest to your profession. If there is, I will bring them here. It is a full set of dental instruments, used in ancient times for the extraction of teeth.

*Dr. Jarvie.*—With reference to the kind offer of Dr. Holcombe, I want to say that this Society has quite a number of interesting specimens of teeth, instruments, and appliances, and we would be very glad to have the instruments the gentleman speaks of. I move you that the instruments be accepted by the Society with thanks to Dr. Holcombe.

The motion was carried.

*Dr. Jarvie.*—The paper read this evening is a very interesting one, and, as Dr. Perry says, contains very few hard words, and is written in a style that every one can understand. I do not suppose there is a gentleman here who could not relate dozens of cases where reflex action from the teeth to the eyes has taken place, but there has been a disposition to deny that affections of the eye ever cause the teeth to ache. About a year ago a lady was brought to me by a physician; she had a splendid set of teeth, but she was suffering from neuralgia in the right side of the face, and she declared the cause must be the first molar. The bicuspids and molars, in fact, all of the teeth on that side of the upper jaw, were perfectly free from decay. Her physician had been treating her for neuralgia for some little time. Occasionally the remedies would have some effect, but generally none at all. I thought possibly the pain might be caused by exostosis or pulpitis, or some obscure trouble, but I could not determine what it was. While the lady located the pain in the first molar, there was nothing to indicate to me that that tooth was the cause of the neuralgia more than any other. About two weeks from the time I first saw her she came to me and insisted upon my extracting that tooth. She was almost demented at the time from pain. Her physician also urged that it be done. Against my better judgment I did extract the tooth, hoping that I might find exostosis or pulpitis, but to my disgust I found no exostosis, no pulp-stones, nothing abnormal about the tooth. The lady went away and her condition remained about the same as before. Her physician was not treating her for any trouble of the eye; whether there was any affection of the eye or not I do not know, but the case was very similar to the one Dr. Stevens has described, and I wish I had heard this paper one year ago.

*Dr. Davenport.*—Mr. President, only a single thought. Our proceedings will be published, of course, as usual, and I think it is hardly just that one statement made by the essayist should be allowed to appear without some reference having been made by a dentist to the changes which have taken place during the last few years in the methods of treating abscessed teeth. Dr. Stevens has told us how unfortunately he was treated when his dentist decided one of his teeth to be in an abscessed condition; and I think any reader of his paper would gather from it that either it was the essayist's opinion, or the opinion of the gentlemen in the dental profession with whom he had associated, that the usual method of treating abscessed teeth which were giving severe pain was to extract them. I do not intend to refer to other methods of treatment, but I wish simply to have it go on record that the cases where abscessed teeth are extracted by the progressive practitioner of the present day are very rare, and then only as a last resort. It may be that in some special cases, such as the one just related by Dr. Jarvie, extraction seems better than to subject the patient to a long course of treatment, the patient being in an anæmic condition, or in a discouraged nervous state from long suffering; but there is a different course to pursue, as we all know, and it is usually well taken.

*Dr. J. Morgan Howe.*—I think we are to be congratulated on the knowledge and interest that the essayist has shown regarding dentistry; although he may have wrongly attributed the reckless practice of former years to the present time. The recognition he has shown of the reflex relations that exist between the teeth and the eye, and also the mutual interest between practitioners who have eyes or teeth under their care, I think is especially a matter for congratulation. We have occasionally in our meetings considered reflex irritations, having their origin in teeth, affecting the ear, and Dr. Houghton, Dr. Sexton, and others have written on that subject with much interest. I think we owe many thanks to the essayist of the evening for having given us the results of his experience, and his recognition of certain mutual reflex disturbances which may be manifested in either eyes or teeth. We must acknowledge that we have been altogether remiss in failing to appreciate the fact that neuralgias so frequently have their origin in ocular disturbance, which may affect the teeth in such a way as to cause us to commit an error. I should say from my present standpoint that the dentist was never—begging our esteemed friend Dr. Jarvie's pardon—justified in extracting a valuable tooth, apparently in good condition, unless he could diagnose a cause for such procedure. We do not know what foolish action the distress and pressure of a patient may lead us into, but in the calm way in which I can speak now I say I would not be justified in taking out a tooth unless I knew there was good reason for such action. I especially wish to thank the essayist for opening up the way for us to dismiss such patients in future by sending them to the oculist.

*Dr. Jarvie.*—I fully agree with Dr. Howe that the case did not justify in cold blood the extraction of the tooth; yet I did it, under the pressure of circumstances existing at that time.

*Dr. J. Bond Littig.*—It will be remembered that I reported a case last year of a patient who suffered for months from severe neuralgia from some cause seemingly remote. I sent that patient to Dr. Stevens, who told him he would need to have an operation performed on his eyes, but the patient did not like the idea and urged me to destroy the pulp of the tooth, which seemed to be a possible cause. The death of the pulp gave no relief, and not until the operation was finally performed on his eyes, two years later, did relief come. If the patient had taken Dr. Stevens's advice at first, he would have had a living pulp in that tooth to-day.

*Dr. Lord.*—Mr. President, I move you that we cast a hearty vote of thanks to the essayist of the evening for his very valuable and instructive paper.

Motion carried.

*Dr. Groat.*—This is a subject that I know very little about, but I have been suffering for three and a half years with neuralgia, and last summer I consulted Dr. Stevens. From the very first operation on the eyes the neuralgic trouble commenced to improve, and I have now very little and only when I use my eyes too much. If I am obliged to operate too long I have pain, but if I am careful of my eyes I have no neuralgia.

*Dr. W. H. Atkinson.*—I am pleased to be permitted to hear the paper and to go over the ground which a competent person has already traversed so clearly. What I rejoice at most is that there is a growing oneness of feeling among professional healers; they are more fraternal, indicating a stronger desire to learn than to be regarded as learned, as formerly. Specialties are not of long standing; and hardly well enough established to find their basis yet; but by continued iteration of the doctrines which have held sway without adequate study, we are constantly in difficulty as to our ability to come to an understanding of the conditions of the sufferers that come into our hands. In the cases detailed before us to-night it is a matter of astonishment to me how it should have been assumed by any one that they were cases of abscess. As to the case in Dr. Stevens's mouth, I did not hear a single word, beyond the pain suffered, that indicated there was anything like a cold or an acute abscess. The way to discriminate an abscess is to observe where the nutrition in the connective tissue is so sluggish as to set up a retrogressive action in the part, and induce the pulp either to die or deposit secondary dentine or pulp-stones, or atrophy, and then, if there be a depleted condition of the general system, the mischief might locate itself at the weak point. We want to see that paper published, then study it carefully, and get what we can out of it, so that we may be able to see eye to eye and help each other in our investigations. There seems to be almost always, when pathological subjects are on the *tapis*, a want of knowledge of basal principles. We forget entirely that wherever there is a nutrient activity being carried forward, in healthy or unhealthy conditions, it always is in that portion of the system where the nutrient changes occur, and that they never do occur except in the soft solids; never in the fluids and never pronouncedly in the hard tissues.

There are a great many recollections in my mind, in a somewhat extended experience, of the difficulties of making examinations and arriving at conclusions, and I know a host of cases such as have been detailed to-night where good sound teeth have been removed with a hope of relieving neuralgia, that finally continued until some operation such as exsection of a nerve-trunk was performed. The trouble with the healers has been that they not only did not know how to help people themselves, but did not know who to consult. Now, thank God, we do know who to send to.

Adjourned.

S. E. Davenport, D.D.S., M.D.S.,
*Editor New York Odontological Society.*

